# A Pilot Study Investigating Changes in Capillary Hemodynamics and Its Modulation by Exercise in the APP-PS1 Alzheimer Mouse Model

**DOI:** 10.3389/fnins.2019.01261

**Published:** 2019-12-04

**Authors:** Xuecong Lu, Mohammad Moeini, Baoqiang Li, Yuankang Lu, Rafat Damseh, Philippe Pouliot, Éric Thorin, Frédéric Lesage

**Affiliations:** ^1^Biomedical Engineering Institute, École Polytechnique de Montréal, Montreal, QC, Canada; ^2^Montreal Heart Institute, Research Center, Montreal, QC, Canada; ^3^Department of Biomedical Engineering, Amirkabir University of Technology (Tehran Polytechnic), Tehran, Iran; ^4^Athinoula A. Martinos Center for Biomedical Imaging, Massachusetts General Hospital, Harvard Medical School, Charlestown, MA, United States; ^5^Department of Surgery, Faculty of Medicine, Université de Montréal, Montreal, QC, Canada

**Keywords:** Alzheimer’s disease, capillary hemodynamics, voluntary exercise, two-photon imaging, neural stimulation

## Abstract

Dysfunction in neurovascular coupling that results in a mismatch between cerebral blood flow and neuronal activity has been suggested to play a key role in the pathogenesis of Alzheimer’s disease (AD). Meanwhile, physical exercise is a powerful approach for maintaining cognitive health and could play a preventive role against the progression of AD. Given the fundamental role of capillaries in oxygen transport to tissue, our pilot study aimed to characterize changes in capillary hemodynamics with AD and AD supplemented by exercise. Exploiting two-photon microscopy, intrinsic signal optical imaging, and magnetic resonance imaging, we found hemodynamic alterations and lower vascular density with AD that were reversed by exercise. We further observed that capillary properties were branch order-dependent and that stimulation-evoked changes were attenuated with AD but increased by exercise. Our study provides novel indications into cerebral microcirculatory disturbances with AD and the modulating role of voluntary exercise on these alterations.

## Introduction

Alzheimer’s disease (AD) is the most common cause of cognitive impairment in the elderly (Iadecola, 2010; Toledo et al., 2013). Dysfunction in neurovascular coupling, resulting in a mismatch between cerebral blood flow and neuronal activity, has long been suggested to play a part in the pathogenesis of AD (Iadecola, 2004, 2010; Zlokovic, 2011; Kisler et al., 2017). AD also shares many risk factors with cardiovascular diseases (Østergaard et al., 2013; Nielsen et al., 2017) and is associated with alterations in vascular function (Iadecola, 2010). Capillaries are the vessels most proximal to neurons (Stefanovic et al., 2008) and thus play a significant role in modulating oxygen extraction to support neuronal activity (Østergaard et al., 2013; Hall et al., 2014; Nielsen et al., 2017). Characterizing microvascular (dys)function in AD is of vital importance, as compromised capillary networks may lead to hypoxia, which, in turn, damages neurons and precipitates Aβ amyloid retention (Nielsen et al., 2017). The first aim of this pilot study was to quantify the changes in capillary red blood cell (RBC) dynamics with AD to provide insights into these issues.

Beyond characterizing hemodynamic dysfunction in microvessels, it is also critical to explore how to mitigate the negative impacts of AD on microcirculation. Physical exercise is a powerful approach for maintaining cognitive health and is hypothesized to play a preventive role against the progression of AD (Radak et al., 2010). Regular aerobic exercise has been found to inhibit the progression of amyloid-related neuropathology in mouse models of AD (Adlard et al., 2005) and to enhance cognitive performance in humans (Pereira et al., 2007; Brown et al., 2010; Hayes et al., 2014). Voluntary exercise has also been shown to improve vascular function in a mouse model of atherosclerosis (Shing et al., 2015). Oxidative stress associated with aging has been found to be reduced with exercise in both humans (Pialoux et al., 2009) and rodents (Durrant et al., 2009). Notwithstanding, the hemodynamic changes in microvascular networks in response to physical exercise with the onset of AD have yet to be studied. Despite a number of hypotheses that indicate the beneficial role of exercise, we still do not have a clear quantification of these changes, which can now be carried out with *in vivo* optical imaging techniques. The second aim of this study was, thus, to quantify the modulatory effect of exercise on cerebral capillaries in AD.

To address these questions, we applied multiple imaging techniques, including two-photon microscopy, intrinsic optical signal imaging (IOSI), and magnetic resonance imaging (MRI), to measure hemodynamic changes at different spatial scales. We found capillary hemodynamic alterations and lower vascular density with AD that were modulated by exercise. We further observed that capillary RBC flow properties were branch order-dependent and that the stimulation-evoked changes were decreased with AD but increased by exercise.

## Materials and Methods

### Animal Groups

Animals were handled according to the Animal Research: Reporting *in vivo* Experiments (ARRIVE) guidelines and the recommendations of the Canadian Council on Animal Care. The protocol was approved by the Ethics Committee of the Research Center of the Montreal Heart Institute. An APP/PS1 mouse model (generous gift from Dr. Bernard Levy, France) and wild-type (WT) mice were genotyped by standard PCR analysis of genomic DNA isolated from ear clips to select both hemizygous APP/PS1 males and their non-transgenic control (WT) male littermates, as previously described (Radde et al., 2006). Mice were kept under standard conditions (24°C; 12-h:12-h light/dark cycle) and were fed *ad libitum* with regular chow (2019S; Harlan Laboratories, Madison, WI, United States). Initially housed four per cage, they were separated and housed one per cage following the surgeries detailed below.

### Experimental Design

Experiments were performed in male wild-type mice at 6 months old (WT6: *n* = 4), transgenic Amyloid Precursor Protein Presenilin-1 (APP/PS1) mice at 6 months old (AD6: *n* = 4), and APP/PS1 mice at 6 months old for which voluntary exercise was initiated at 3 months old (AD6&EX: *n* = 4). Previous work showed a clear onset of cognitive symptoms in APP/PS1 mice at 4.5 months old (Cifuentes et al., 2015) and established at 6 months old (de Montgolfier et al., 2019), and thus, we conducted imaging at 6 months old and investigated the hemodynamic changes in this relatively early stage of AD. For the AD6&EX group, exercise was voluntary. Mice in the exercise group were housed individually from 3 months old forward in separate cages to which a running wheel was attached with a rotation counter. Mice started running on wheels at 3 months old and continued the daily exercise until 6 months old, when imaging data were recorded. The wheel (diameter 15 cm, recorded by an easyMATRIX 16 EMKA) was mounted outside the home cage, and the total number of wheel rotations was recorded daily on an LE907 individual counter. The running distance averaged across mice ranged from ∼2 to ∼4 km per day in the 3-month exercising period.

**FIGURE 1:**
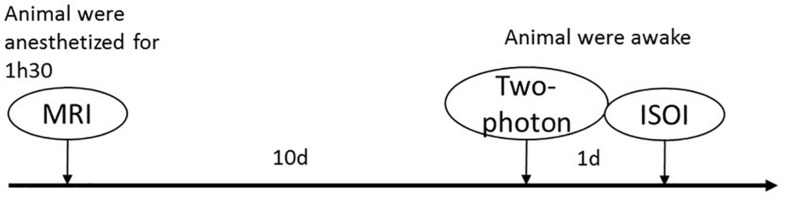
Timeline of imaging experiments.

Multiple imaging techniques were used for the data collection, as shown in Figure 1. The order of procedures was as follows. At 6 months old, mice were imaged with MRI in anesthetized (1h30, isoflurane) condition. Ten days later, two-photon imaging was conducted to collect capillary properties in awake animals. One day later, intrinsic signal optical imaging (ISOI) was performed to collect data on changes in hemoglobin, again in awake animals. Each mouse was included in all of these experiments.

### Two-Photon Imaging

For two-photon imaging, a craniotomy was performed, and mice were familiarized with the awake imaging device. On the day of cranial surgery, animals were anesthetized with isoflurane (1.5–2.0% in pure oxygen), with their physiological parameters monitored in real time. A fixation bar was attached for awake imaging. At the same time as bar fixation, a cranial imaging window was prepared to allow for two-photon imaging of the cortex following well-established guidelines (Shih et al., 2012). A cranial window with a 3-mm diameter was made over the left barrel cortex (0.5 mm posterior to bregma, 3.5 mm lateral to the midline). Agarose in artificial cerebral spinal fluid (ACSF) was placed over the exposed skull, and then a three-layer cover glass was glued onto the skull to generate a transparent cranial window. Ketoprofen (5 mg/kg, Merial, Canada) and buprenorphine (0.05 mg/kg, Reckitt Benckiser Healthcare, United Kingdom) were injected before the surgery for analgesic and antipyretic effects. Baytril (5 mg/kg, Bayer, Germany) was injected after the surgery and repeated one day after the surgery. To adapt to the device during imaging, animals were trained on the imaging wheel in four fixation-training sessions beginning after 3 days of recovery following the cranial surgery. The length of fixation time gradually increased from 10 to 45 min over the four sessions.

A home-built two-photon microscope was used to record functional capillary data and vascular density. The MaiTai-BB laser oscillator (Newport Corporation, United States) of the two-photon microscope generated a sequence of 820 nm, 80 MHz, 150 fs pulses modulated by an acousto-optic modulator for power control. The fluorescent dye (dextran-FITC) was injected through the tail vein to distinguish plasma (in bright colors) and RBCs (appearing as dark shadows) in the recorded images. The line scans were performed both perpendicularly and longitudinally to each measured capillary over 250-ms time intervals to generate space-time images. In consecutive longitudinal scans, the dark shadows move toward the right or left of the scanning beam because of the movement of RBCs, resulting in tilted dark streaks in the space-time image (Kleinfeld et al., 1998). The angle of these dark streaks was used to quantify RBC velocity, with streaks closer to horizontal orientation associated with higher velocity. In perpendicular line scans, the signal is dark when an RBC is passing through the optical focus, and RBC flux can be assessed. Following line scanning, 3D angiograms were obtained over four overlapping 600 × 600 μm (400 × 400 pixels) regions at depths of 100–550 μm with 5-μm steps at a frame rate of 0.5 Hz. During all measurements, animals were awake, with the head fixed by a titanium bar and limbs free to move on the rotating wheel (Moeini et al., 2018, 2019).

### Measurement of Capillary Properties

The spacetime images were used to obtain the following capillary parameters, following (Moeini et al., 2018): (1) diameter, by fitting the perpendicular scans with a Gaussian function with estimated diameter at half maximum of the distribution; (2) RBC velocity from the angle of the streaks in longitudinal scanning; (3) RBC flux, calculated by the number of dark shadows divided by the acquisition time of the images (the flux was obtained from the average of values in longitudinal and perpendicular scans); and (4) hematocrit, calculated by RBC flux × RBC volume/capillary volumetric flow [capillary volumetric flow = RBC velocity × capillary cross-sectional area; RBC volume assumed to be 55 μm^3^ for C57Bl/6 mice (Wirth-Dzięciołowska et al., 2009)]. The angiogram images were used to compute the vascular density using a deep-learning segmentation approach based on the FC-Densenets architecture (Damseh et al., 2018a, b). The vascular density was quantified with each slice of the segmented two-photon angiograms by restricting the analysis to vessels with a diameter of less than 8 um.

We also divided the capillaries based on branching order. The artery region, with the diving arteriole, precapillary arteriole, and capillaries, was labeled with respect to branching order A1, A2, and A3, with A1 branching off directly from the precapillary arterioles. The vein region, with the surfacing venule and post-capillary venule, was labeled with the branching order V1, V2, and V3, with V1 linking directly to veins. In our recordings, the capillary segments ranged from 6 to 12 branch orders. We only labeled and defined the first three branch orders from arterioles (A1, A2, and A3) and venules (V1, V2, and V3). When a capillary path had more than six branching orders, these additional branch orders were merged into V3, thus enabling consistent numbering for all capillaries observed.

### Magnetic Resonance Imaging (MRI)

Anatomical and perfusion MRI scanning was performed with a 30 cm 7T horizontal MR scanner (Agilent, Palo Alto, CA, United States) before two-photon imaging. The mice were in a prone position and imaged with a gradient insert coil (12-cm inner diameter, gradient strength 600 mT/m, and rise time 130 ms). The brain was imaged with a 2-channel receive-only surface coil and a quadrature transmit/receive birdcage coil (69-mm internal diameter) (RAPID Biomedical, Germany), while the animal was anesthetized with 1.4–2.2% isoflurane in air with 30% oxygen, with the body temperature kept at 37.0°C using a warm air fan (SA Instruments, Stony Brook, NY, United States). Respiration was monitored with a target of ∼100 bpm, adjusting the level of isoflurane only whenever the rate was outside 80–120 bpm. Heart rate was monitored with a pulse oximeter.

Anatomical images were recorded with a 3D true free induction with steady-state precession (TFISP) sequence (Bowen et al., 2006) at a 100-μm isotropic resolution (TR = 5.0 ms, TE = 2.5 ms, 16 frequencies, 22-min scan time). A 3D amplitude-modulated continuous arterial spin labeling scan (amCASL) was then acquired (TR = 3.0 s, labeling duration = 1.0 s, 60 × 54 × 48 matrix, 300 μm × 333 μm × 333 μm resolution, 23-min scan time).

The anatomical recordings were first aligned manually to a previously produced anatomic template using ITK-SNAP and then co-registered non-linearly with the advanced normalization tools (ANTs) (Avants et al., 2010, 2011), thus producing the ANT transformations to co-register the perfusion scans to a common referential. Perfusion was calculated voxel-by-voxel as described in Chugh et al. (2012), with assumed parameters of brain/blood partition coefficient = 0.9 mL/g, mouse arterial blood transit time = 0.08 s, tagging efficacy = 0.67, T_1__b_ = 2.3 s, T_1_ = 1.53 s, T_1__sat_ = 0.57 s, and M_a_^z^(w) = 0.48 s. The MarsBar toolbox in SPM was also used to extract the average perfusion data (Delafontaine-Martel et al., 2018).

### Intrinsic Signal Optical Imaging (ISOI)

An ISOI system (Labeo Technologies, Inc.) was used to investigate the stimulus-evoked hemodynamic responses in all experimental groups. Whiskers of mice were deflected at a rate of 10 Hz to activate the somatosensory cortex, with a 5-s stimulation time and a 15-s post-stimulus period. Illumination using green and red lights was positioned to illuminate the exposed skull of the mouse head. Images were acquired by a camera at a rate of 5 Hz per wavelength over a period spanning 10 stimulation repetitions. We computed changes in oxy-hemoglobin concentration (Δ[HBO](t)), deoxy-hemoglobin concentration (Δ[HBR](t)), and total hemoglobin concentration (Δ[HBT](t)), as was done in Dubeau et al. (2011a, b).

### Statistical Analysis

The results are presented as mean ± SEM. Non-parametric Kruskal–Wallis tests were applied with follow-up tests for multiple group comparisons. Statistical significance was assigned at ^+^*p* < 0.1, ^∗^*p* < 0.05, ^∗∗^*p* < 0.01, ^∗∗∗^*p* < 0.001.

## Results

### Capillary RBC Flow Is Altered by AD and Modulated by Exercise

To better understand the role of exercise and AD on capillary RBC flow properties, we assessed the values of the resting capillary diameter, RBC velocity, RBC flux, and capillary hematocrit in all groups and their coefficient of variation (CV) using two-photon microscopy.

**FIGURE 2:**
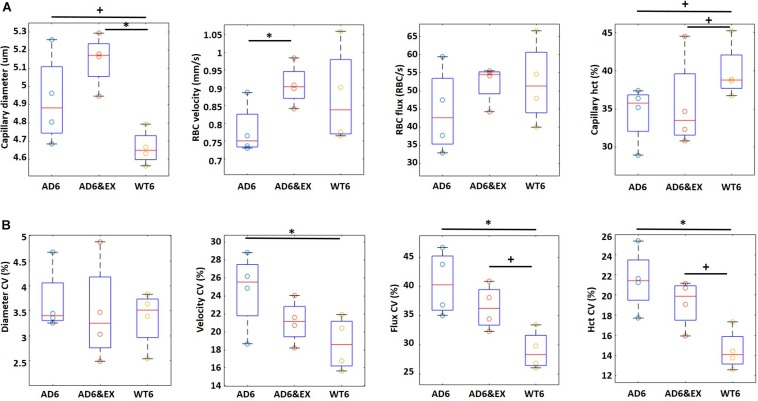
Capillary RBC flow characteristics in different groups (four mice per group). **(A)** Capillary diameter, RBC velocity, flux, and hematocrit in different experimental groups. **(B)** Coefficient of variation (CV) for all capillary parameters. The CV in each capillary was computed based on multiple frames recorded each 0.25 s within a 5-s time window. Thus, CV represents the temporal variations of each property within a capillary. CV was computed as the standard deviation divided by the mean. ^∗^*p* < 0.05 and ^+^*p* < 0.1.

In Figure 2A, we observed that the capillary diameter had an increasing trend from the WT6 group (4.67 ± 0.06 μm) to the AD6 group (4.98 ± 0.07 um, *p* = 0.09) and significantly increased from the WT6 group to the AD6&EX group (5.15 ± 0.08 μm, *p* = 0.011). A decreasing trend of RBC velocity from WT6 (0.87 ± 0.03 mm/s) to AD6 (0.79 ± 0.03 mm/s) was also observed (see Figure 2A). RBC velocity increased significantly with exercise (velocity in the AD6&EX group: 0.91 ± 0.04 mm/s, *p* = 0.04, Figure 2A). However, there were no significant differences in RBC flux between groups (Figure 2A). Finally, the capillary hematocrit had a decreasing trend from the WT6 group (39.57 ± 1.15%) to the AD6 group (35.15 ± 1.30%, *p* = 0.09) (see Figure 2A). Exercise elevated the hematocrit (AD6&EX group, 37.32 ± 1.19%), as expected from physiology (see Figure 2A).

Given the hypothesis that capillary heterogeneity is a key element in oxygen delivery, we also computed the CV of these parameters. The CV represents the variations of each property within a capillary as a function of time. In Figure 2B, we show the CV of each parameter to explore the influence of AD and exercise on capillary heterogeneity. We observed significant variations in the velocity CV, flux CV, and hematocrit CV (see Figure 2B), while we did not find significant differences regarding the diameter CV in different groups. The velocity CV decreased significantly from the AD6 group (24.63 ± 1.92%) to the WT6 group (18.69 ± 1.33%, *p* = 0.049). The flux CV increased (*p* = 0.01) from the WT6 group (29.17 ± 1.25%) to the AD6 group (40.63 ± 2.13%) (see Figure 2B). Exercise trended to decrease the flux CV from AD6 to AD6&EX (35.50 ± 1.58%) (see Figure 2B). Finally, hematocrit CV increased from WT6 (14.65 ± 0.79%) to AD6 (20.95 ± 1.41%, *p* = 0.008), while exercise trended toward a reduction of the hematocrit CV from AD6 to AD6&EX (18.42 ± 1.08%, see Figure 2B). Taken together, the capillary velocity flux and hematocrit were reduced with AD, associated with increased variations over time, while exercise regularized parameters toward values found in WT mice. Capillary diameter was the exception to this. Given the increased heterogeneity in RBC velocity, RBC flux, and hematocrit with AD, we also analyzed the variations of these properties as a function of branch order of capillaries to explore how they varied from the upstream to the downstream segments. The results of this analysis are provided in the Appendix.

### High Temporal Fluctuations in Capillary RBC Velocity and Flux Were More Frequent With AD

**FIGURE 3:**
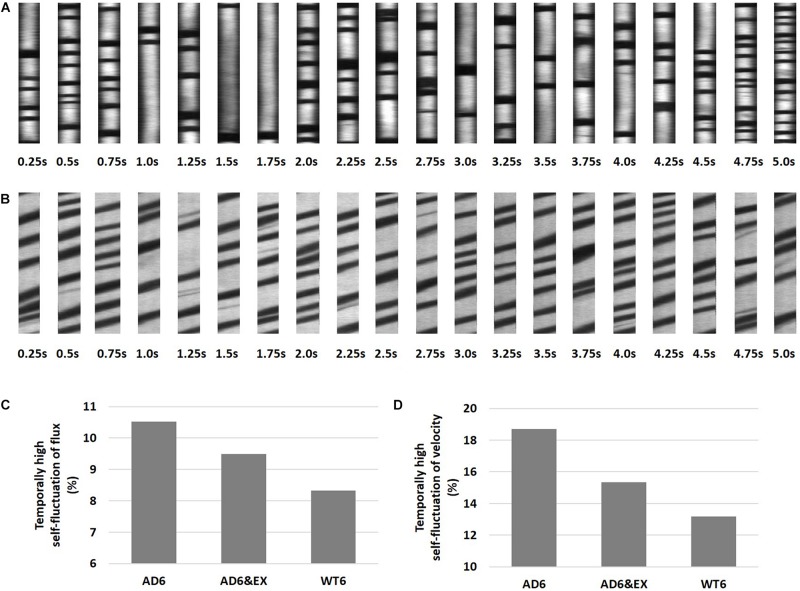
High temporal capillary fluctuations of RBC velocity and RBC flux. **(A)** Representative space-time images with higher fluctuations of RBC flux as a function of time, with 0.25 s intervals during a 5-s recording window. The flux was obtained using both longitudinal and perpendicular scans, and the average value was used. **(B)** Longitudinal equivalent RBC velocity was calculated from the angle of dark streaks in a specific recording frame. **(C)** The percentage of capillaries with high temporal fluctuations of RBC velocity in all groups. The “isoutlier” method in MATLAB was applied to detect outliers with a standard deviation of RBC velocity exceeding three standard deviations from the mean. **(D)** Same for RBC flux.

Previous studies observed spontaneous stalls in capillary RBC flux in individual capillary segments (Erdener et al., 2017; Hernández et al., 2019). We thus analyzed our data to capture the presence of highly fluctuating RBC properties in capillaries by quantifying the fraction of capillaries with outlier standard deviations of RBC flux or speed within a 5-s recording window (see Figures 3A,B). Although stalling was not characterized in our experiments, these capillary outliers tend to display highly fluctuating flux and velocity, which may influence oxygen extraction (Gutiérrez-Jiménez et al., 2016; Østergaard et al., 2016). Examples for capillary outliers with temporally high self-fluctuation of RBC flux and velocity were shown in Figures 3A,B, respectively.

We further calculated and compared the fraction of outliers in all groups and found that the proportion with high temporal self-fluctuating RBC velocity was highest in the AD6 group (10.43%, 48 outliers in 460 capillaries), followed by the AD6&EX group (9.45%, 48 outliers in 508 capillaries), and was lowest in the WT6 group (8.19%, 46 outliers in 562 capillaries) (see Figure 3C). We did the same analysis for RBC flux (see Figure 3D). We again found that the proportion with high temporal self-fluctuating RBC flux was highest in the AD6 group (18.70%, 86 outliers in 460 capillaries), followed by the AD6&EX group (15.35%, 78 outliers in 508 capillaries), and was lowest in the WT6 group (13.17%, 74 outliers in 562 capillaries) (see Figure 3D).

### Higher Vascular Density Peak Value and Lower Average Vascular Density in the AD Group

**FIGURE 4:**
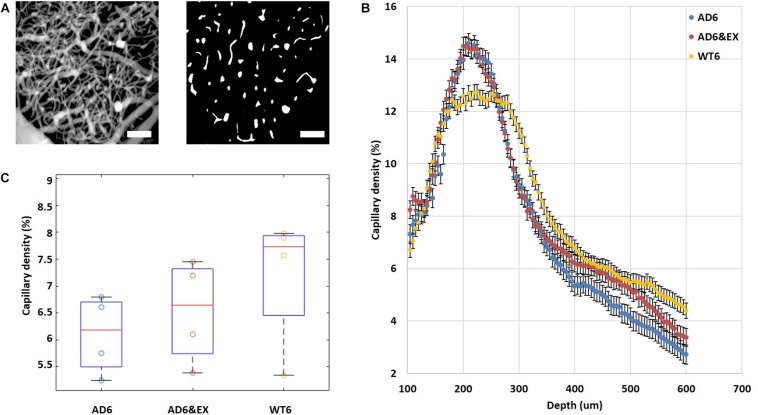
Vascular distribution in different experimental groups and at different depths. **(A)** Binarization of microvascular angiograms was applied to calculate the vascular density. Left: MIP image with depths ranging from 100 to 550 μm under the brain surface with 5 μm steps. Right: an example of a binary segmentation of a single *en face* slice after removing the large horizontal vessels at a depth of 120 um (scale bar: 100 μm). **(B)** Average vascular density in different experimental groups as a function of depth from 100 to 550 μm under the brain surface. **(C)** Estimated vascular density (volume%). Results are presented as box plots with the median value (red line).

We calculated the vascular density from two-photon angiograms (see Figure 4A) after binary segmentation. Interestingly, all experimental groups reached their peak values of vascular density at a depth of ∼220 μm and the values then gradually decreased with increasing depth beneath the brain surface (see Figure 4B). The AD groups (both AD6 and AD6&EX) had significantly higher peak values (AD: 14.60 ± 0.98%; AD6&EX: 14.48 ± 1.09%) when compared with the peak value of the WT6 group (12.74 ± 0.70%) (see Figure 4B). Voluntary exercise did not significantly change the peak value of vascular density (see Figure 4B). When integrating across depths, the AD6 group showed a trend toward a lower average value (8.16 ± 0.37%) compared to the WT6 group (8.85 ± 0.29%) (see Figure 4C). With voluntary exercise, the AD6&EX group (8.64 ± 0.34%) exhibited a trend of higher vascular density than the AD6 group (see Figure 4C). Overall, higher vascular density peak values and lower average vascular densities were observed in the AD groups.

### AD Reduced Brain Perfusion and Task-Related Changes, With the Latter Modulated by Exercise

**FIGURE 5:**
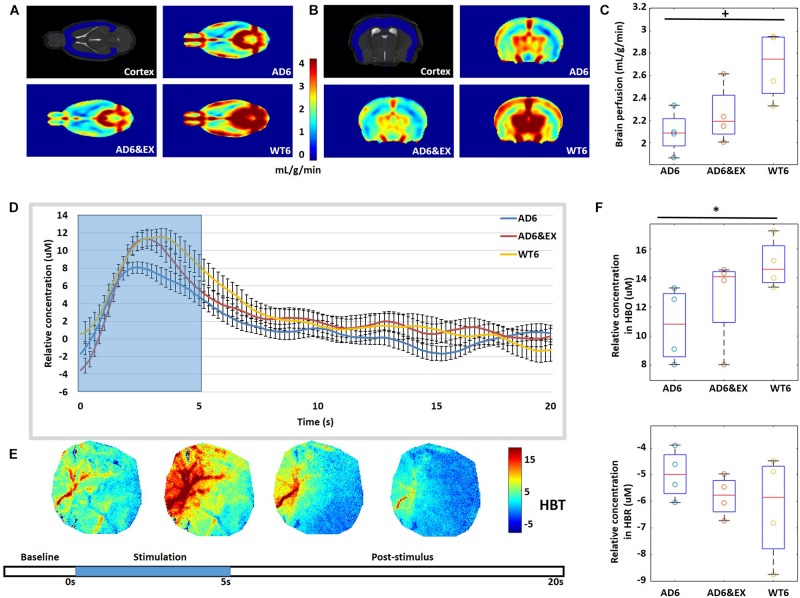
**(A,B)** Typical 7-T MRI images, with coronal and axial views of the cortex. **(C)** Quantification of cerebral perfusion (ml/g/min) by MRI. **(D)** Averaged temporal response of oxy-hemoglobin (HBO) for all groups with 5-s stimulation (blue background). **(E)** Temporal dynamics of total hemoglobin (HBT) response to whisker stimulation. Stimulation lasted for 5 s, followed by a 15-s rest. **(F)** Averaged results for change in HBO (Δ[HBO](t)) and change in HBR (Δ[HBR](t)) over all mice. ^∗^*p* < 0.05 and ^+^*p* < 0.1.

We used MRI to quantify brain perfusion in each group (see Figures 5A,B). We observed that brain perfusion decreased from the WT6 group (2.69 ± 0.15 ml/g/min) to the AD6 group (2.16 ± 0.10 ml/g/min, *p* = 0.06) (see Figure 5C). However, exercise did not significantly modulate brain perfusion in AD groups (AD6&EX: 2.13 ± 0.16 ml/g/min) (see Figure 5C).

Intrinsic signal optical imaging was also used to investigate the response to stimulation to investigate task-related changes in hemoglobin concentration (see Figures 5D,E). The whiskers of mice were deflected to activate the somatosensory cortex, with a 5-s stimulation time and a 15-s post-stimulus period. We observed increased HBO with stimulation for all groups, while HBO had higher peak values in the WT6 group and the AD6&EX group when compared to the AD6 group, as shown in Figure 5D. We found that the Δ[HBO](t) in the AD6 group (10.75 ± 0.64 μM) was lower (*p* = 0.03) than that in the WT6 group (15.00 ± 0.85 μM). However, exercise tended to increase the Δ[HBO](t) from the AD6 group to the AD6&EX group (12.69 ± 0.85 μM, see Figure 5F upper panel). Finally, there were no significant differences regarding Δ[HBR](t) between groups (see Figure 5F lower panel).

## Discussion

In this study, we aimed to quantify the hemodynamic properties of and modulatory role of exercise on cerebral capillaries in AD due to the important role of microcirculation in cognitive health (Østergaard et al., 2016; Erdener et al., 2017; Zhang et al., 2017; Hernández et al., 2019). Exploiting awake microscopic vascular imaging and a transgenic mouse model of AD, we found that in this model (APP-PS1), AD is associated with hemodynamic disruption in capillaries. Voluntary exercise was seen to decrease capillary hemodynamic heterogeneity, although exercise did not increase the global cerebral blood flow in the cortex.

### Capillary Flow Property Changes Due to AD and Exercise

Blood flow was slightly reduced in the AD6 group compared with WT6 mice but tended to increase with exercise in the AD6&EX group compared with the AD6 group. Specifically, RBC velocity tended to decrease with AD, with moderate modulation by exercise. The disrupted RBC velocity could result from the occlusion of vessels due to amyloid deposition (Arbel-Ornath et al., 2013). In another study (Lu et al., 2019), we gathered immunohistological evidence showing increased amyloid deposition with AD with a moderate, but significant, reduction due to exercise. Future research may consider immunohistological markers such as inflammation to shed light on the impact of AD on capillary properties more directly. The reduced RBC velocity could, in turn, lead to compromised perfusion in the mouse cortex, further slowing the clearance of amyloid and increasing its deposition (Kisler et al., 2017). We further observed that hematocrit was reduced with AD compared with the WT mice, with an increasing trend with exercise. A previous study (Moeini et al., 2018) found reduced hematocrit in healthy aging, thus disrupting capillary oxygen delivery efficiency. The prior literature has also observed miscrocirculation flow disruption in other cardiovascular diseases, such as stroke (Kazmi et al., 2013) and hypertension (Pialoux et al., 2009), and in even healthy aging (Dubeau et al., 2011b). The microvascular flow alterations observed in our study suggest that AD does share many similar microvessel disruptions with cardiovascular pathologies and aging. These microvessel problems could be alleviated by exercise, as is the case in other cardiovascular diseases (Pialoux et al., 2009; Brown et al., 2010).

We also found that RBC properties, especially velocity and flux, were branch order-dependent for all experimental groups. Prior study (Sakadžić et al., 2014) suggests that oxygen is in good proportion extracted in the first few branches after the precapillary arteriole in normal physiological conditions. Our results were related to previous findings and showed that RBC velocity and flux decreased from the upstream capillaries to the downstream capillaries for all groups to allow for oxygen delivery. Capillaries are responsible for transporting oxygen and nutrients to brain tissue and clearing metabolic end products from the brain to venous circulation (Kisler et al., 2017). In another paper (Lu et al., 2019), we found that indeed, tissue oxygen was reduced and more heterogeneous in mice with AD, consistent with this study. In capillary segments close to arterioles, capillaries were observed to have larger RBC velocity and flux, potentially enabling the delivery of oxygen and energy metabolites to the brain tissues. Future research may further explore the functioning of different capillary branches and investigate whether AD could lead to differentiated impacts on these segments.

Capillary obstruction due to flow dysfunction and tissue inflammation has been observed in animal models and has been shown to contribute to the development of neuron pathology and disease (Santisakultarm et al., 2014; Erdener et al., 2017; Hernández et al., 2019). Inflammation and flow disruption are well-recognized features of AD (Gaugler et al., 2018). Inflammation triggers reactive oxygen species, which lead to obstruction in capillaries (Park et al., 2008). Meanwhile, amyloid deposition may also lead to vessel occlusion by disrupting the interstitial fluid drainage pathway (Arbel-Ornath et al., 2013). Although we only focused on non-stalling capillaries in this study, the highly fluctuating RBC velocity and flux could indicate potential microvascular flow problems. We found a higher fraction of capillaries with high self-fluctuations of RBC velocity and flux in AD, slightly decreased with exercise. Previous research suggests that capillary flow patterns should homogenize to support metabolic demands, whereas heterogenous velocities across the capillary network diminish the oxygen extraction capabilities (Stefanovic et al., 2008; Gutiérrez-Jiménez et al., 2016). Our results suggest more heterogeneous flow distribution in the AD group when compared to WT but a reduced heterogeneity in the AD&EX group compared with the AD group, which may underly the benefits of exercise in AD.

### Vascular Morphology Alterations in AD

Studies on morphological abnormalities of capillaries in AD have reported lower capillary densities due to deposition of Aβ amyloid (Kitaguchi et al., 2007). Previous work indicated a more rapid loss of vascular density with age, leading to compromised cerebral perfusion (Farkas and Luiten, 2001). Our results indicate that AD is associated with lower vascular density, which could contribute to reduced cerebral perfusion. This should be set against other studies that showed spatial and age-related variations in these vascular density differences between WT and APP/PS1 mice (Delafontaine-Martel et al., 2018). Here, the observed reduction in the somatosensory cortex could be potentially mitigated by exercise. Several previous studies have demonstrated the capacity of exercise to improve cognitive function (Hamer and Chida, 2008; Durrant et al., 2009; Mekari et al., 2015). Here, our data partially support the positive role of voluntary exercise on the maintenance of healthy vascular density. One intriguing observation was that at a depth of 200 um, the vascular density was higher in AD mice than in controls. The vascular density in this AD model (and others) is age-dependent, and previous work has documented higher vascular density in AD mice when compared to WT (Cifuentes et al., 2015) in the cortex but not in the hippocampus. This indicates a potential complex distribution of vascular changes that cannot be accounted for by a single local observation. AD could also influence capillary diameter constriction and dilation, which respond to brain demands (Iadecola, 2017). Prior studies have shown that pericytes, the multi-functional cells wrapping around the capillaries, are able to influence capillary diameter to adjust oxygenation levels (Østergaard et al., 2013; Iadecola, 2017). Minimal changes in capillary diameter could produce large changes in oxygen delivery, since capillaries are closest to neurons in the vascular network and have large surface areas. Interestingly, we observed increased capillary diameters in the AD group and the exercise group. This change in diameters could be due to the compromised flow in AD groups, with the diameters dilating to compensate for the hypoperfusion. Another possible reason for the dilation of capillaries could be the occlusion of capillaries in AD due to amyloid deposition or inflammation (Park et al., 2008; Arbel-Ornath et al., 2013), as occlusion of vessels was shown to lead to vessel dilation in upstream capillaries in stroke disease (Nguyen et al., 2011).

### Reduced Cerebral Perfusion and Functional Responses in AD

Using ISOI, we measured the functional hemodynamic response to stimulation (Sheth et al., 2005). Prior studies used ISOI to investigate pathological departures of neurovascular coupling in diseases such as focal epileptic seizures that exhibit an elevation in HBO (Schwartz et al., 2011). Another study applied ISOI and observed that functional connectivity strength was reduced in patients with AD, in cognitively normal elderly with elevated amyloid deposition, and in advanced aging (Bero et al., 2012). Our results corroborate previous findings showing a lower response amplitude in AD, suggesting neuro-vascular coupling changes. In contrast, the HBO response further increased with voluntary exercise, suggesting that exercise may increase the regional oxygen delivery efficiency in capillaries but not through global increases in CBF. This finding was supported by MRI results suggesting decreased overall cerebral perfusion in both AD groups. Beyond the mesoscopic hemodynamic response to stimulation, future studies may consider characterizing the hemodynamic response to whisker stimulation at the capillary level directly using two-photon microscopy.

### Limitations of the Study

The major limitation of our study is the small sample size used for the analyses, which may lead to potential type I (characterized as false-positive results) and type II (characterized as false-negative results) errors (Hackshaw, 2008). We admit that the associations we found were from a hypothesis-generating study, and a larger confirmatory study is needed in future. Additionally, the individual housing condition of the AD&EX group may cause some extent of social deprivation, which may mitigate the benefits of exercise. Future studies may need to find a balance between the social inclusion factors and the validity of voluntary exercise.

## Conclusion

Our study provides novel insights into the hemodynamic mechanisms of cerebral microcirculatory disturbances in AD and the modulatory effects of voluntary exercise on these hemodynamic alterations. Our work suggests therapeutic targets that may alleviate microvascular complications observed in AD.

## Data Availability Statement

The datasets generated for this study are available on request to the corresponding author.

## Ethics Statement

Animals were handled according to the Animal Research: Reporting *in vivo* Experiments (ARRIVE) guidelines and the recommendations of the Canadian Council on Animal Care. The protocol was approved by the Ethics Committee of the Research Center of the Montreal Heart Institute.

## Author Contributions

XL designed the study, carried out the experiments, analyzed the data, and drafted the manuscript. MM and PP carried out the experiments. MM, BL, YL, RD, and PP analyzed the data. ET participated in the designing of the study. FL conceived, designed, and coordinated the study, and revised the manuscript. All authors gave final approval for publication.

## Conflict of Interest

FL reports a minority ownership in LabeoTech, Inc. The remaining authors declare that the research was conducted in the absence of any commercial or financial relationships that could be construed as a potential conflict of interest.

## Figures and Tables

**FIGURE A1:**
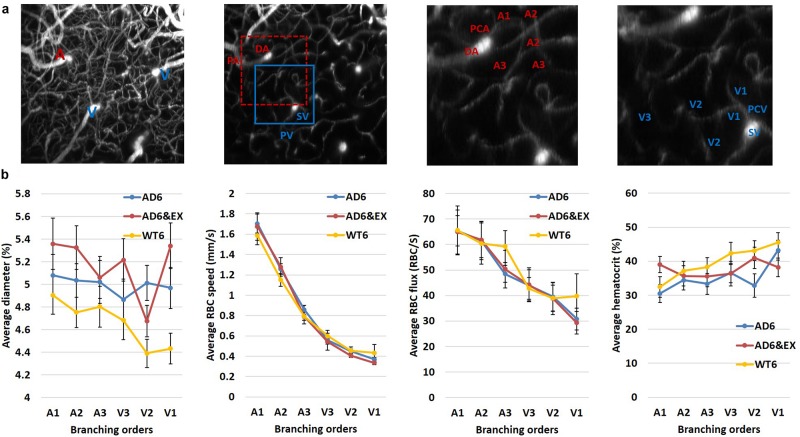
Capillary property changes with branching orders. **(a)** Left: a maximum intensity projection image of the angiogram. Second from the left: an *en face* slice at the depth of ~100 μm. The red square indicates the area used to calculate branching orders from arterioles, and the blue square indicates the area used to calculate branching orders from venules. Third from left: the enlarged artery region with diving arteriole, precapillary arteriole, and capillaries labeled with branching orders. Fourth from left : the enlarged vein region with surfacing venule and postcapillary venule, labeled with the branching orders. **(b)** The changes of capillary diameter/velocity/flux/hematocrit as a function of branching order (460, 508, and 562 capillaries measured in the AD6, AD6&EX, and WT6 groups, respectively). **(c)** Diameter/velocity/flux/hematocrit differences among the first three branching orders in different groups (460, 508, and 562 capillaries measured in the AD6, AD6&EX, and WT6 groups, respectively).

## Appendix

### Branch Dependent Investigation of Capillary RBC Properties

We used the 3D angiogram to distinguish different branching orders in capillaries (see Appendix Figure 1a). We investigated how capillary hemodynamics varied in different branching orders for all groups.

We observed that the average diameter showed a slight decreasing trend in WT6 group, while its value remained stable in the AD6 group (see Appendix Figure 1b first from the left). With exercise, the AD group remained relatively stable with a dip in the downstream capillaries (see Appendix Figure 1b first from the left). The smaller diameter in downstream capillaries could be linked to the stalling occuring more in the downstream capillaries (Erdener et al., 2017). The average RBC velocity showed sharp decrease in the upstream capillaries for all groups (see Appendix Figure 1b second from the left). The average RBC flux also had sharp reduction in all branches for the AD groups, but had a moderate decrease in the WT group (see Appendix Figure 1b third from the left). Finally, all groups showed increasing trends of hematocrit from upper to lower streams (see Appendix Figure 1b the fourth from the left).

Due to the larger reduction in the first three branching orders of capillary RBC velocity and flux, we further calculated the RBC property differences among the initial three orders for each group to explore the differences across branches. We observed that for the initial three orders (from A1 to A3), the RBC velocity dropped at least 62% [calculated by (A1-V3)/A1] (67.28, 67.70, and 62.25% for AD6, AD6&EX, and WT6, respectively), suggesting that the RBC velocity was largely modulated in the upper stream of capillaries. Additionally, for the average RBC flux, we observed that in the initial three orders (from A1 to A3), the flux dropped ~ 32% over three groups [calculated by (A1-V3)/A1] (32.74, 31.87, and 34.83% for AD6, AD6&EX, and WT6, respectively), indicating that the RBC flux modulation in the upper orders still largely contributed to the variance of overall RBC flux across all capillaries.
